# Optimizing myelin water imaging in multiple sclerosis

**DOI:** 10.1016/j.nicl.2026.104080

**Published:** 2026-11-02

**Authors:** Ellis Donders, Guus H.J. Vorst, Pien M. Schipper, Joost P.A. Kuijer, Eva M.M. Strijbis, Joep Killestein, Hugo Vrenken, Frederik Barkhof, Menno M. Schoonheim, Petra J.W. Pouwels

**Affiliations:** a MS Center Amsterdam, Amsterdam UMC location Vrije Universiteit Amsterdam, Department of Radiology and Nuclear Medicine, De Boelelaan 1117, Amsterdam, the Netherlands; b Amsterdam Neuroscience, Brain Imaging, Amsterdam, the Netherlands; c Amsterdam Leukodystrophy Centre, Department of Child Neurology, Emma Children's Hospital, and Amsterdam Neuroscience, Amsterdam University Medical Center, Vrije Universiteit, Amsterdam 1105, AZ, the Netherlands; d MS Center Amsterdam, Amsterdam UMC location Vrije Universiteit Amsterdam, Department of Neurology, De Boelelaan 1117, Amsterdam, the Netherlands; e Amsterdam Neuroscience, Neuroinfection & -inflammation, Amsterdam, the Netherlands; f MS Center Amsterdam, Amsterdam UMC location Vrije Universiteit Amsterdam, Department of Anatomy and Neuroscience, De Boelelaan 1117, Amsterdam, the Netherlands; g Queen Square Institute of Neurology, Queen Square, London WC1N 3BG, United Kingdom

**Keywords:** Myelin water fraction, Multiple sclerosis, Demyelination, Multi-echo gradient echo, Multi compartment relaxometry

## Abstract

Myelin water imaging (MWI) allows in vivo quantification of myelin content using MRI. Multi-echo gradient-echo (MEGRE) sequences with Variable Flip Angles (VFA), combined with diffusion-weighted imaging (DWI), can generate robust, whole-brain myelin water fraction (MWF) maps more efficiently than conventional implementations. We evaluated how spatial resolution and number of flip angles affect MWI quality in a clinical research setting.

Fifteen people with MS and ten healthy controls were scanned at 3 T (Siemens VIDA). MRI included multi-shell DWI and 3D-MEGRE acquired at 2.0 mm and 2.5 mm isotropic resolution. Twelve echoes were acquired at five flip angles. MWF was quantified based on a three-compartment T1, T2*, and DWI model, and determined in normal appearing white matter (NAWM), lesions and corpus callosum. Associations between MWF across resolutions and VFA schemes were assessed using Bland-Altman, ICC-C and ICC-AA.

MWF was highest in corpus callosum (10.2 ± 1.1%), intermediate in NAWM (7.9 ± 0.7%), and lowest in lesions (4.9 ± 0.7%) (values for 2.0 mm and FA5). A FA4 MEGRE protocol showed comparable MWF values, while FA3 systematically overestimated MWF and showed greater variability, particularly in lesions. ICC-C between spatial resolutions was good to excellent (0.80–0.91), ICC-AA was moderate to good (0.66–0.81), indicating that the two resolutions should not be treated interchangeably. QC monitoring of fitting results is recommended to strengthen reliability of MWF results.

MWI duration can be shortened with a FA4 MEGRE protocol. Resolution choice should be guided by study aims, as 2.0 mm and 2.5 mm were not fully interchangeable.

## Highlights

• Multi-compartment relaxometry enables myelin water imaging in multiple sclerosis.
• Whole-brain myelin water imaging at 2.0 mm resolution takes under 6 min.
• A faster 2.5 mm scan gives higher, but not directly comparable, myelin water fractions.
• Myelin water fraction is lower in lesions than in normal-appearing white matter.
• Accurate quality control of voxelwise model fits is important to ensure reliability.

## 1 Introduction

Multiple sclerosis (MS) is a chronic neuroinflammatory disease with focal lymphocytic infiltration leading to damage of myelin and axons, and primary neurodegenerative processes that contribute independently to tissue damage (Filippi et al., 2018). This damage can disrupt neuronal communication across many systems, ranging from cognitive impairments to motor function. Magnetic resonance imaging (MRI) is essential for diagnosis and disease monitoring in MS (Rovira et al., 2015); but conventional MRI primarily provides qualitative information and cannot quantify tissue microstructural properties. Accurate quantification of the extent of myelin damage and repair is important for monitoring the effect of targeted disease modifying therapies.

Myelin water imaging (MWI) is considered a reliable technique for imaging myelin content in vivo (Laule et al., 2006). The central principle behind MWI is that water associated with different microstructural environments in the brain, such as myelin sheaths, intra−/extracellular space and cerebrospinal fluid, exhibit distinct relaxation properties. By decomposing the overall MR signal into components with different relaxation properties, MWI allows for the selective quantification of the “myelin water” signal fraction, which reflects water trapped between the lipid layers of the myelin sheaths (MacKay and Laule, 2016). The myelin water fraction (MWF) is the amount of water in this myelin compartment relative to the total water signal, and has been shown to correlate with histologically measured myelin content (Laule et al., 2006). Previous studies have demonstrated that MWF is sensitive to abnormalities not only within MS lesions but also in normal-appearing white matter (NAWM) (Allen et al., 2001), and that it relates to downstream neurodegeneration such as cortical atrophy (Kolind et al., 2015). Early MWI techniques were based on 2D acquisitions, which typically provided only one or a few thicker slices and required long scan times. The multi-echo gradient echo (MEGRE) method, especially when combined with multi-shell diffusion-weighted imaging (DWI), has shown good performance in terms of clinical correlations, homogeneity, and reproducibility (Chan and Marques, 2020). Moreover, it was demonstrated in a small group of healthy volunteers that the MEGRE method can be shortened without compromising data quality (Chan et al., 2023), thereby reducing scan time and opening new possibilities for implementation in MS research and clinical practice. Despite these advances, the application of robust whole-brain MWI in MS remains limited, and its potential to differentiate lesions and NAWM in a clinically feasible timeframe has not yet been fully established.

In this study, we aim to implement and evaluate the MEGRE-based MWI method in combination with multi-shell DWI in a clinical research setting of people with MS (pwMS) and healthy controls. Our primary aim is to investigate how spatial resolution and number of flip angles affect MWI quality, and whether acquisition time can be shortened without compromising the sensitivity of the resulting MWF maps. As MWF is considered a surrogate measure of myelin content, we expect it to be lower in lesions than in NAWM, and lower in NAWM of pwMS than in white matter of healthy controls.

## 2 Methods

### 2.1 Demographics

This prospective study was approved by the local Ethics Committee, and participants provided written informed consent. Twenty participants from the PrograMS cohort (Noteboom et al., 2023) were prospectively enrolled between October and December 2025, including 15 pwMS and 5 age-matched healthy controls (HC). The pwMS had a diagnosis of Relapsing Remitting MS (RRMS), Primary Progressive MS (PPMS) or Secondary Progressive MS (SPMS) based on the 2017 McDonald Criteria (Thompson et al., 2018). An additional 5 HCs who had previously undergone the identical MRI protocol outside of this study period were retrospectively included for comparison, yielding a total study population of 25 participants (15 pwMS, 10 HC).

### 2.2 MRI data acquisition

All MR scans were performed on a 3 Tesla MR scanner (Vida, Siemens Healthcare GmbH, Erlangen, Germany). A 64-channel head coil was used for 22 of the 25 participants; in the remaining 3 cases a 20-channel coil (with larger diameter) was used to accommodate a larger head size. The MR protocol included a sagittal 3D T1-weighted magnetization-prepared rapid gradient echo (MPRAGE) sequence (echo time TE = 2.32 ms, repetition time TR = 2300 ms, inversion time TI = 900 ms, acquired voxel size = 0.9 × 0.9 × 0.9 mm^3^, flip-angle = 8°) and a sagittal 3D fluid-attenuated inversion recovery (FLAIR) sequence (TE = 388 ms, TR = 5000 ms, TI = 1650 ms, acquired voxel size = 1 × 1 × 0.9 mm^3^). A sagittal 3D multi-echo gradient-echo (3D-MEGRE) sequence was acquired at two isotropic spatial resolutions (2.5 mm and 2.0 mm). In both cases, a CAIPIRINHA acceleration with a factor of 5 was used. Each acquisition used 12 echoes and five flip angles (10°, 20°, 30°, 40°, and 70°). At 2.5 mm isotropic resolution, the imaging parameters were TR/TE₁/ΔTE/TE₁₂ = 32.5/1.56/2.94/30.93 ms, with an acquisition time of 50 s per flip angle, resulting in a total scan time of 4 min 10 s. At 2.0 mm isotropic resolution, parameters were TR/TE₁/ΔTE/TE₁₂ = 33.9/1.70/2.94/32.28 ms, and the acquisition time was 88 s per flip angle, yielding a total scan time of 7 min 20 s. Multi-shell diffusion-weighted imaging (DWI) was acquired with single spin-echo echo-planar imaging in an oblique transverse plane, aligned parallel to the anterior–posterior commissure (AC–PC) line, with 2.5 mm isotropic spatial resolution. The protocol consisted of 6 b = 0 images, 16 diffusion-weighted images at b = 1000 s/mm^2^, and 32 diffusion-weighted images at b = 2000 s/mm^2^. Diffusion-encoding gradient directions were optimized and uniformly distributed using MRtrix3 (Tournier et al., 2019). To allow correction for susceptibility-induced geometrical distortion, we acquired two volumes with reversed phase-encoding and without diffusion. A B1 field map was acquired (TR/TE   =  9500/1.9 ms, flip angle   =  8°), at low spatial resolution (8.1 × 8.1 × 6.0 mm^3^).

### 2.3 DWI data processing

Multi-shell DWI data underwent distortion and motion correction using FSL's topup and eddy tools (FMRIB's Software Library, version 6.0.4, Oxford, UK), with eddy configured to perform outlier replacement (Andersson et al., 2003) (Andersson and Sotiropoulos, 2016). Probabilistic fiber tracking preparation was conducted using BedpostX to estimate the fiber orientations that are used by the Multi-Compartment Relaxometry-Diffusion Informed MWI (MCR-DIMWI) analysis (Hernandez-Fernandez et al., 2019). NODDI modeling utilized the Watson distribution model and was implemented via the CUDA Diffusion Modeling Toolbox (version 9.1, Oxford, UK). Diffusion-derived fiber orientation and volume fraction maps were registered from native DWI space to MEGRE space using affine transformation, at the spatial resolution of the corresponding MEGRE protocol (2.0 mm or 2.5 mm). Three quantitative NODDI metrics were derived: neurite density index (NDI), orientation dispersion index (ODI), and isotropic volume fraction (FISO) (Zhang et al., 2012). Diffusion tensor imaging (DTI) metrics were calculated from the b-values of 0 and 1000 s/mm^2^ using DTIFIT.

### 2.4 MWF analysis

MEGRE data was processed according to the MCR-DIMWI pipeline, using a three-compartment model of intra-axonal, extra-axonal and myelin water, based on T1 and T2* relaxometry. White matter fiber orientation and volume fractions of the compartments from diffusion data were used in the model as well. We used the model assumptions and all fixed parameters as described by Chan et al. (Chan et al., 2023) and Stellingwerff et al. (Stellingwerff et al., 2025). Flip-angle volumes were linearly co-registered to FA20 using ANTs (Stellingwerff et al., 2025), based on alignment of the first echo of each flip angle to the first echo of FA20. Gibbs unringing was applied to remove ringing artifacts (Billot et al., 2023), and phase images were shifted so that the median phase of the FA20 first-echo (brain-masked) image was centered at zero. Flip angles were adjusted voxelwise, using the B1 map that was linearly registered to the FA20 first-echo image as well. In the MCR-DIMWI fitting process we used quadratic weighting, as outlined by Chan et al. (Chan et al., 2023). A voxelwise goodness-of-fit is given by the residual, which represents the difference between the actual signal intensity of all echo times and flip angles and the signal intensity of the model fit, where a lower residual indicates a better fit. To make this quality control measure independent of the number of echo times and flip angles, the residuals were normalized by dividing by the number of flip angles and the number of echo times used in the analysis and multiplying by 100 (Stellingwerff et al., 2025). Voxels with residuals exceeding a threshold of the median plus 3 times the interquartile range (IQR) within the brain mask were considered unreliable and were excluded from the analyses (Stellingwerff et al., 2025). To get insight into the distribution of normalized residuals within regions of interest (ROIs) in the full cohort, we determined the 90th percentile within each ROI (after the exclusion of voxels above the median + 3IQR limit). This value was subsequently used as a lower threshold to visualize voxels with relatively low fit quality in individual MWF maps.

To evaluate the impact of reducing acquisition time, MWF maps were reconstructed using three flip angle combinations: the full FA5 dataset (10, 20, 30, 40, 70°), a FA4 subset (10, 20, 40, 70°), and a FA3 subset (10, 20, 70°), based on previous research (Chan et al., 2023). Because the FA5 protocol was the most complete acquisition protocol, all subsets were compared against it as reference.

### 2.5 Regions of interest and lesions

Automatic brain segmentation of 3D T1 was performed using SynthSeg (version 7.4.1) (Billot et al., 2023) and visually inspected. Additionally, automatic lesion segmentation of 3D T1 and 3D FLAIR was performed using LST-AI (Wiltgen et al., 2024). The lesions were manually corrected where necessary. NAWM masks were obtained using the white matter segmentation from SynthSeg followed by lesion masking. DTI toolkit version 2.3.1 (Wang et al., 2007) was used to register DWI brains to a common space, allowing registration of atlas-based ROIs. Using ANTs, ROIs defined in T1 space were transformed into MEGRE space (first echo of flip angle 20 magnitude image) and diffusion space (FA map) via affine registration. The ROIs were based on the JHU atlas and included occipital, parietal, frontal and temporal lobes, the cerebellum and corpus callosum (Zhang et al., 2006). For each ROI and subject, the median and IQR for each quantitative measure were extracted.

### 2.6 Statistical analysis

All analyses were performed in RStudio software (version 4.4.3; R Foundation for Statistical Computing, Vienna, Austria). Group-level statistics are subsequently reported as the mean ± standard deviation of the per-subject medians across participants, and all analyses were performed on these group means. Agreement in MWF between flip-angle protocols (FA3, FA4, FA5) within each spatial resolution, and between spatial resolutions (2.0 mm, 2.5 mm) within each flip-angle protocol was assessed using Bland-Altman analysis, reporting the mean difference (bias) and 95% limits of agreement (mean difference ± 1.96 SD). MWF estimates across resolutions were assessed using the intraclass correlation coefficient for consistency (ICC-C) and absolute agreement (ICC-AA), reported with 95% confidence intervals. The ICC-AA assesses exact agreement; the ICC-C disregards systematic difference between measurements. The ICC outcomes were interpreted using the standards of Koo and Li (2016) (Koo and Li, 2016). Associations between MWF and diffusion-weighted metrics were assessed using Pearson correlation coefficient. For all subsequent analyses, MWF estimates from the 5 flip angle, 2.0 mm protocol were used as the reference standard. Associations between MWF and continuous clinical variables (lesion volume, disease duration) were assessed using Pearson correlation coefficients. Differences in MWF across MS subtypes (RRMS, SPMS, PPMS) were evaluated using the Kruskal-Wallis test, and considered exploratory given the small and unequal group sizes. Where the Kruskal-Wallis test was significant, post-hoc pairwise comparisons were performed using the Wilcoxon rank-sum test with Bonferroni correction. Group differences between pwMS and HCs were assessed using independent samples *t*-tests, and within-subject differences between tissue compartments (NAWM vs lesions) using paired *t*-tests. A significance threshold of *p* < 0.05 was applied throughout.

## 3 Results

### 3.1 Demographics

The demographics of the included pwMS and controls are described in Table 1. One patient with MS (male, SPMS) was excluded from the analysis due to MRI artifacts (see 3.5 Quality Control), leaving 24 participants (14 pwMS, 10 HC) in the final analysis.

**Table 1:** Demographics and Clinical Characteristics.

Characteristics	Controls	All patients	RRMS	PPMS	SPMS
Subjects, N	10	14	7	4	3
Age, y, median, (IQR)	50.9 (33.5–59.1)	60.6 (54.6–68.8)	58.3 (50.5–60.2)	69.6 (65.5–70.7)	62.6 (61.3–64.4)
Sex F/M	6/4	8/6	4/3	2/2	2/1
Disease duration since onset, y, median, (IQR)	N/A	26.5 (23.5–38.7)	24.7 (22.4–26.5)	35.2 (21.0–47.5)	37.3 (33.4–38.2)
Lesion volume, mL (IQR)	N/A	10.2 (6.7–15.0)	9.9 (6.9–10.9)	16.4 (10.2–27.5)	8.2 (5.7–16.2)

**Note.** Abbreviations; RRMS: Relapsing Remitting MS; PPMS: Primary Progressive MS; SPMS: Secondary Progressive MS.

### 3.2 Myelin water imaging parameters

Fig. 1 shows axial MWF maps across different resolutions and flip angle combinations for one subject with MS. Axial, sagittal, and coronal views of the full 3D whole-brain MWF maps are provided in Supplementary Fig. A.1.

**Fig. 1:**
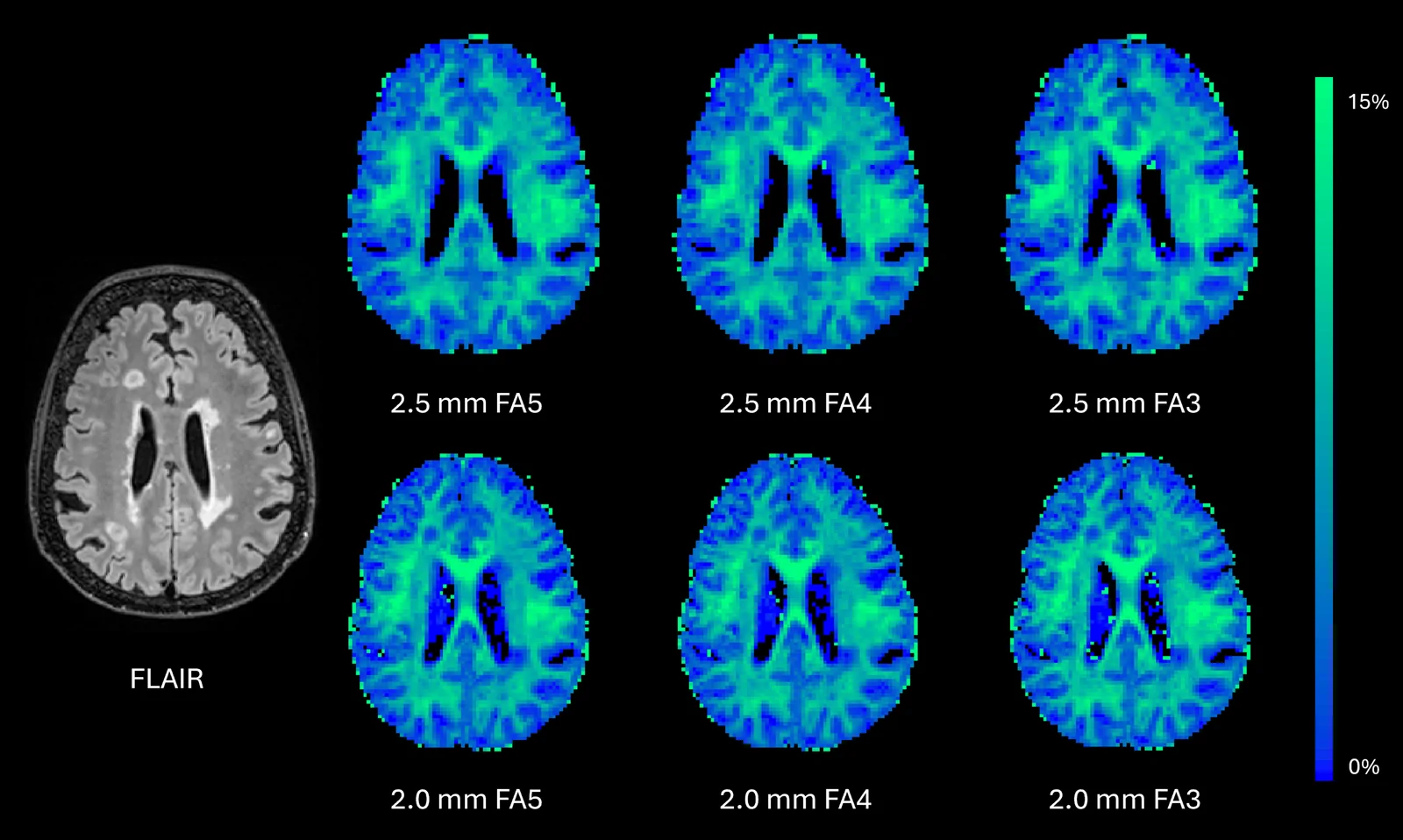
Axial FLAIR and MWF maps of a female with SPMS (age 60.9 years; disease duration 37.3 years). There is large similarity between MWF maps obtained at different resolutions and number of flip angles.

Table 2 shows MWF values across different ROIs, resolutions, and flip angle combinations. Using a spatial resolution of 2.0 mm and FA5 as example, mean MWF was highest in the corpus callosum (10.2 ± 1.1%), intermediate in NAWM (7.9 ± 0.7%), and lowest in lesions (4.9 ± 0.7%). This pattern was observed across all flip angle combinations and resolutions, and consistent with the expected pattern of myelin content across these tissue types. Analyses based on FA3 had higher MWF values compared to both FA4 and FA5, while FA4 and FA5 produced similar values. This pattern was consistent across both resolutions and all three ROIs examined. The close agreement between FA4 and FA5, and the overestimation of MWF with FA3 is illustrated in scatter plots in Fig. 2, for values in NAWM. Similar scatter plots for MWF values in corpus callosum and lesions can be found in Supplementary Figs. A.2 and A.3, respectively. MWF values obtained with 2.5 mm resolution were typically higher than at 2.0 mm. The variability in MWF values between two spatial resolutions was slightly larger than the variability between protocols with different numbers of flip angles at a fixed resolution, as shown in scatter plots in Fig. 3.

**Table 2:** MWF values across different flip angle combinations and resolutions.

ROI	Resolution	FA3	FA4	FA5
NAWM	2.0 mm	8.3 ± 0.7%	8.0 ± 0.7%	7.9 ± 0.7%
	2.5 mm	9.1 ± 0.9%	8.6 ± 0.8%	8.5 ± 0.9%
Corpus Callosum	2.0 mm	10.8 ± 1.2%	10.3 ± 1.1%	10.2 ± 1.1%
	2.5 mm	11.5 ± 1.2%	10.8 ± 1.2%	10.8 ± 1.0%
Lesions	2.0 mm	5.2 ± 0.8%	5.0 ± 0.7%	4.9 ± 0.7%
	2.5 mm	6.0 ± 1.1%	5.6 ± 0.9%	5.6 ± 0.9%

**Note.** NAWM and corpus callosum are mean MWF values over all participants. Lesions are mean MWF values over all pwMS.

**Fig. 2:**
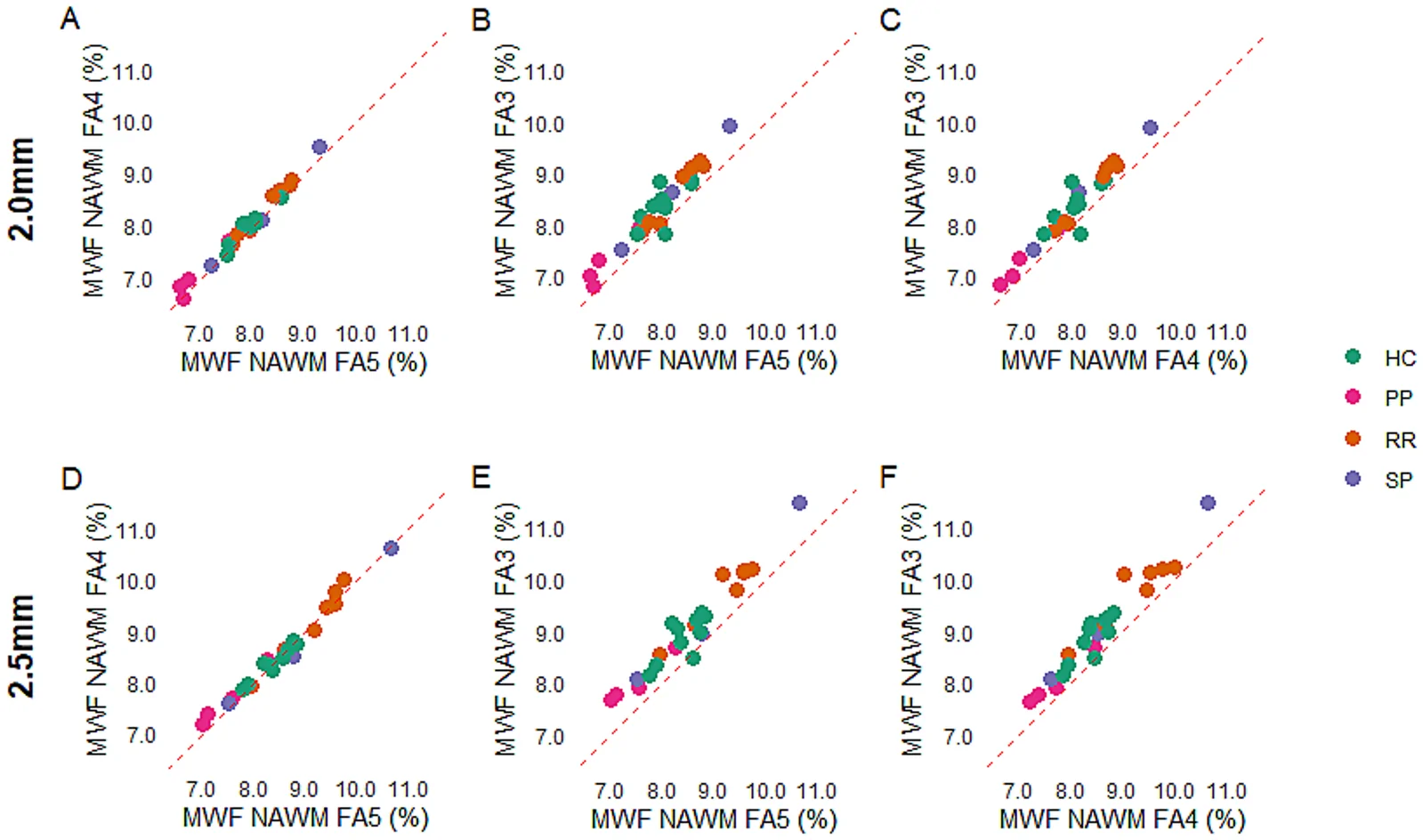
Scatter plots showing MWF values within NAWM across protocols with different number of flip angles. Panels depict: FA4 vs. FA5 at 2.0 mm (A), FA3 vs. FA5 at 2.0 mm (B), FA3 vs. FA4 at 2.0 mm (C), FA4 vs. FA5 at 2.5 mm (D), FA3 vs. FA5 at 2.5 mm (E), and FA3 vs. FA4 at 2.5 mm (F). Abbreviations: MWF, myelin water fraction; NAWM, normal appearing white matter; FA, flip angle.

**Fig. 3:**
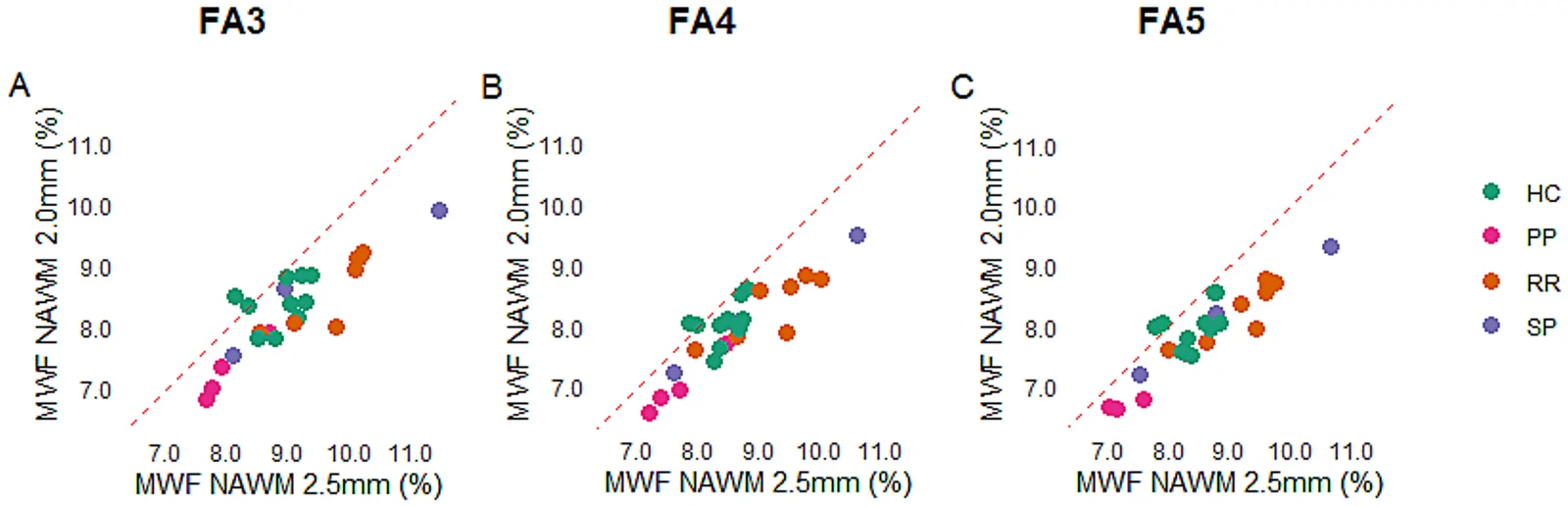
Scatter plots showing MWF values within NAWM across different resolutions. Showing 2.0 mm vs 2.5 mm for FA3 (A), 2.0 mm vs 2.5 mm for FA4 (B), 2.0 mm vs 2.5 mm for FA5 (C).

Bland-Altman analysis of MWF in NAWM showed systematic differences between flip-angle protocols at both resolutions (Fig. 4). At 2.0 mm, FA3 showed a positive bias relative to FA4 (mean difference 0.32%, LoA −0.08 to 0.72%) and relative to FA5 (0.39%, LoA −0.05 to 0.82%), whereas FA4 showed minimal bias relative to FA5 (0.06%, LoA −0.13 to 0.26%). A similar pattern was observed at 2.5 mm, with larger biases for FA3 versus FA4 (0.48%, LoA 0.03–0.93%) and FA3 versus FA5 (0.53%, LoA 0.08–0.98%), while FA4 versus FA5 again showed the smallest bias (0.05%, LoA −0.21 to 0.31%) and narrowest limits of agreement. Figures demonstrating the systematic differences in corpus callosum and lesions are provided in the supplementary materials (Fig. A.4 and A.5, respectively).

**Fig. 4:**
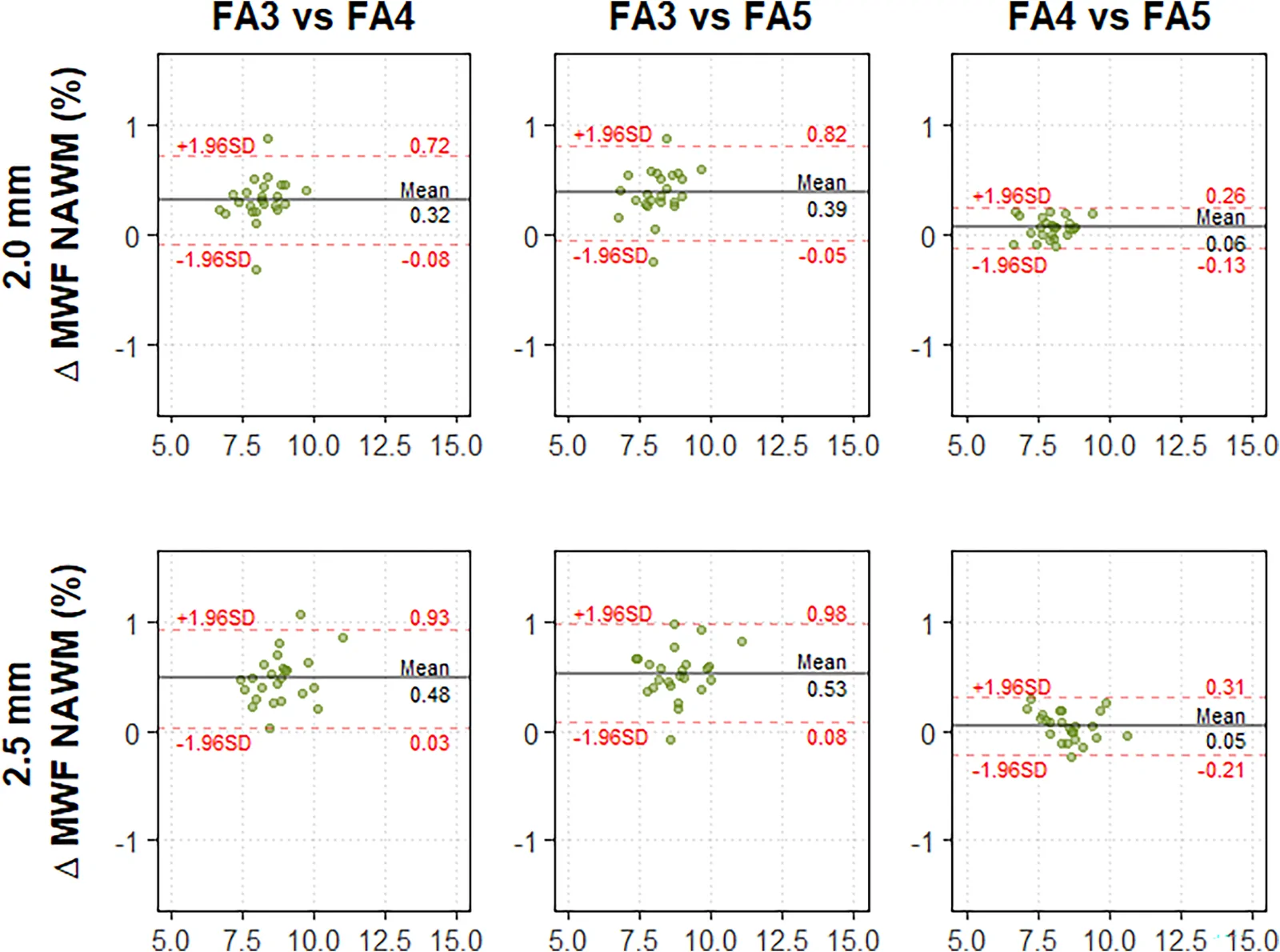
Bland-Altman plots comparing MWF estimates in NAWM across flip angle protocols at two spatial resolutions.

Fig. 5 shows the Bland-Altman analysis of MWF in NAWM revealing systematic differences between spatial resolutions at each flip-angle protocol. Across all three protocols, 2.0 mm MWF values were systematically lower than 2.5 mm values, with the largest bias observed for FA3 (mean difference − 0.76%, LoA −1.66 to 0.15%). This bias was slightly smaller for FA4 (−0.60%, LoA −1.38 to 0.18%) and FA5 (−0.61%, LoA −1.41 to 0.18%), which showed nearly identical mean differences and limits of agreement. Similar systematic differences were observed for corpus callosum and lesions (Supplementary Figs. A.6 and A.7, respectively).

**Fig. 5:**
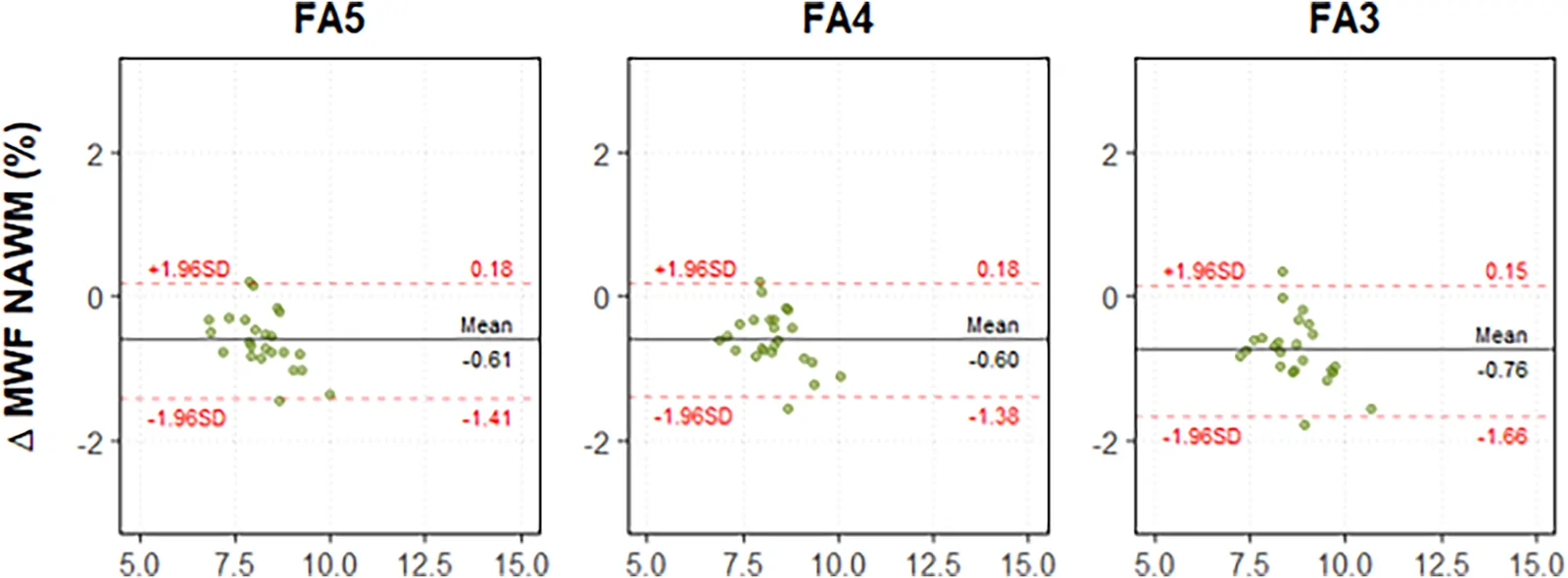
Bland-Altman plots comparing MWF estimates in NAWM across spatial resolutions at three flip angle protocols. ΔMWF = MWF(2.0 mm) – MWF (2.5 mm).

Fig. 6 shows the ICC-C and ICC-AA between resolutions. The FA4 and FA5 protocols yielded good to excellent ICC-C values between resolutions across all ROIs (NAWM: 0.86, corpus callosum: 0.91, lesions: 0.91), while the FA3 protocol showed good but slightly lower ICC-C values, particularly for lesions (ICC-C: 0.80, 95% CI: 0.48–0.93), in accordance with the scatter plots of Fig. 3. In contrast to the high ICC-C values, ICC-AA was moderate to good and consistently lower across all ROIs (NAWM: 0.60–0.67, corpus callosum 0.74–0.82, lesions 0.58–0.68) and flip-angle protocols, reflecting the systematic bias identified in the Bland-Altman analysis. This divergence between ICC-C and ICC-AA confirms that while MWF values are consistently ranked across resolutions, they are not directly interchangeable in absolute terms, particularly for NAWM and lesions.

**Fig. 6:**
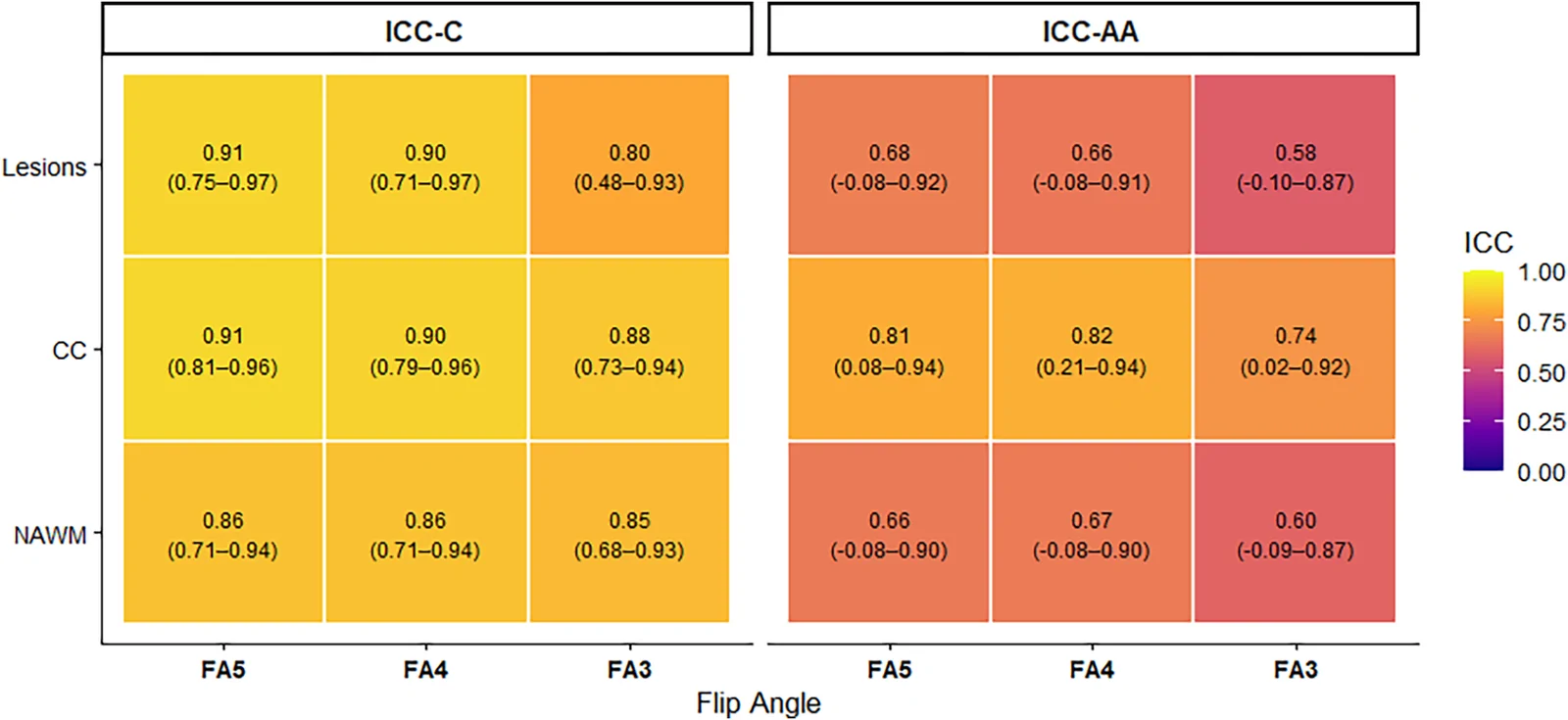
Heatmap of ICC-C (consistency) and ICC-AA (absolute agreement) scores between resolutions with the 95% confidence intervals, for different ROIs.

To assess whether the model fit quality differed between resolutions, normalized residuals were compared between 2.0 mm and 2.5 mm acquisitions across all ROIs. No significant differences in residuals were observed in NAWM (*p* = 0.742), corpus callosum (*p* = 0.101), or lesions (*p* = 0.617), indicating that the three-compartment model fit the data equally well at both resolutions. Furthermore, when tested on the FA5 protocol, mean residuals did not differ significantly between resolutions in either HC (2.0 mm: 0.0133 ± 0.0062 vs. 2.5 mm: 0.0128 ± 0.0052; *p* = 0.846) or pwMS (2.0 mm: 0.0168 ± 0.0060 vs. 2.5 mm: 0.0179 ± 0.0060; *p* = 0.091). Normalized residuals per flip angle and resolution can be found in Supplementary Table A1.

### 3.3 MWF in pwMS and HC

In pwMS, NAWM MWF (7.9% ± 0.8%) was significantly higher than lesion MWF (4.9% ± 0.7%), *p* < 0.001. No significant differences were observed between pwMS and healthy controls in either NAWM MWF (7.9% ± 0.8% vs 8.0% ± 0.3%; *p* = 0.56) or corpus callosum MWF (10.2% ± 0.6% vs 10.1% ± 1.4%; *p* = 0.74). In an exploratory analysis of MS subtypes, for which the subgroup sizes were small (RRMS *n* = 7, PPMS *n* = 4, SPMS *n* = 3), pairwise comparisons revealed that PPMS had significantly lower NAWM MWF compared to both RRMS (6.9% ± 0.4% vs 8.2% ± 0.5%; *p* = 0.002) and HC (6.9% ± 0.4% vs 8.0% ± 0.3%; *p* = 0.008), whereas no significant differences were observed between RRMS and SPMS or between SPMS and PPMS (Fig. 7). A similar pattern was observed for corpus callosum MWF, where PPMS again showed the lowest values, though this did not reach statistical significance (*p* = 0.055). Lesion MWF did not differ significantly across subtypes (*p* = 0.19). Given the small subgroup sizes and the older age of PPMS participants relative to RRMS in this cohort, these subtype findings should be interpreted as preliminary. Greater lesion volume was associated with lower NAWM MWF (*r* = −0.55, *p* = 0.041), lower corpus callosum MWF (*r* = −0.68, *p* = 0.008), and lower lesion MWF (*r* = −0.70, *p* = 0.006). Neither disease duration nor age showed significant associations with MWF in any ROI (disease duration: all *p* > 0.57; age: NAWM *r* = −0.12, *p* = 0.590; corpus callosum *r* = −0.01, *p* = 0.959; lesions *r* = 0.1, *p* = 0.737). All analyses were done on FA5 and 2.0 mm resolution.

**Fig. 7:**
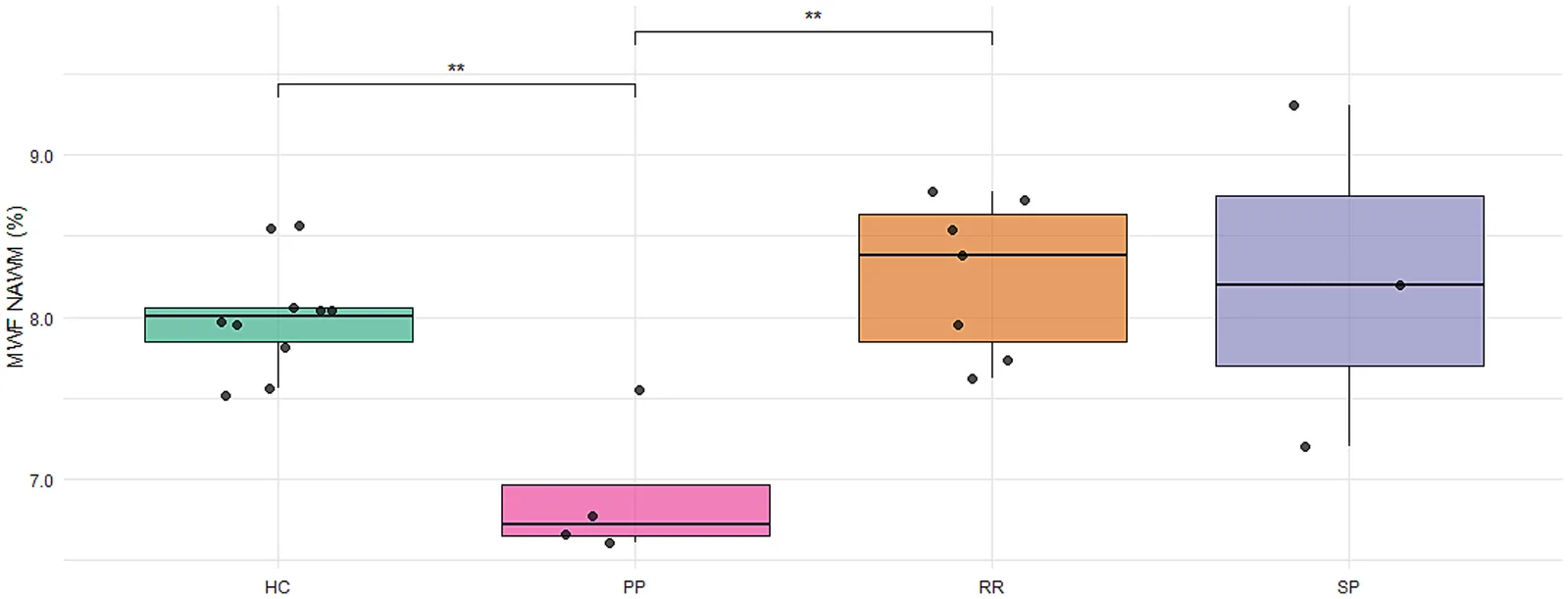
NAWM MWF (%) across groups. Boxplots display the distribution of myelin water fraction (MWF) in normal-appearing white matter (NAWM) for healthy controls (HC) and the three MS subtypes: primary progressive (PPMS), relapsing-remitting (RRMS), and secondary progressive (SPMS). Significant pairwise differences are indicated by brackets (** *p* < 0.01). Analysis done using 5 flip angles at 2.0 mm resolution.

### 3.4 Associations with diffusion MRI metrics

The association between NAWM MWF and diffusion MRI metrics was assessed using Pearson correlations across the total study population (pwMS and HC combined) across four MWF acquisition protocols (2.5 mm FA4, 2.5 mm FA5, 2.0 mm FA4, 2.0 mm FA5). Results were highly consistent across FA protocols within each resolution (Table 3). At 2.0 mm resolution, several diffusion metrics showed statistically significant associations with MWF. Fractional Anisotropy and NDI were positively correlated with MWF (Fractional Anisotropy: *r* = 0.46–0.49, *p* < 0.05; NDI: *r* = 0.50–0.52, *p* < 0.05), while AD, RD and MD showed significant negative correlations (AD: (*r* = −0.42, *p* < 0.05; RD: *r* = −0.46 to −0.48, *p* < 0.05; MD: *r* = −0.49 to −0.51, *p* < 0.05). At 2.5 mm resolution, none of the diffusion metrics showed statistically significant correlations with MWF (all *p* > 0.14), despite the direction and magnitude of associations being largely consistent with the 2.0 mm findings. The latter suggests that the lack of significance at 2.5 mm is likely attributable to reduced sensitivity rather than a true absence of association. ODI showed no significant associations with MWF at either resolution.

**Table 3:** Correlations between MWF and Diffusion Metrics in NAWM for different resolutions and number of flip angles.

	2.5mm FA5	2.5mm FA4	2mm FA5	2mm FA4
FA	0.30 (p=0.150)	0.31 (p=0.141)	0.46* (p=0.023)	0.49* (p=0.015)
AD	-0.21 (p=0.315)	-0.22 (p=0.296)	-0.42* (p=0.040)	-0.42* (p=0.042)
MD	-0.25 (p=0.245)	-0.26 (p=0.215)	-0.49* (p=0.015)	-0.51* (p=0.012)
RD	-0.22 (p=0.302)	-0.24 (p=0.259)	-0.46* (p=0.025)	-0.48* (p=0.016)
NDI	0.30 (p=0.155)	0.31 (p=0.136)	0.50* (p=0.013)	0.52** (p=0.009)
ODI	-0.20 (p=0.338)	-0.20 (p=0.360)	-0.21 (p=0.325)	-0.24 (p=0.259)

**Note.** Abbreviations; FA: Fractional Anisotropy; AD: Axial Diffusivity; MD: Mean Diffusivity; RD: Radial Diffusivity; NDI: Neurite Density Index; ODI: Orientation Dispersion Index.

### 3.5 Quality control

In all participants, there was signal loss at later echoes in areas with B0 inhomogeneity, leading to higher residuals in these areas. Based on outliers per measurement per subject, these areas were removed, as described in the methods and illustrated in Supplementary Fig. A.8. These areas were primarily located in cortical areas such as orbitofrontal and temporal. On average, for the FA5 datasets, only 2.2% of NAWM volume was excluded due to residuals higher than the threshold (range 0.9 – 3.3% at 2.0 mm and 1.0–3.8% at 2.5 mm). The mean percentage of included voxels in the NAWM ROI did not differ between resolutions (97.8 ± 0.7% for 2.0 mm, and 97.9% ± 0.6% for 2.5 mm). Normalized residuals within the retained tissue were evaluated across all participants, and mean values were between 0.015 and 0.025 in corpus callosum and NAWM, as shown in Table A.2. The 90th percentile of normalized residuals within ROI was typically around 0.05. This threshold of 0.05 was chosen to visualize voxels with relatively low fit quality. Based on this quality control, one participant was excluded from all analyses due to consistently high normalized residuals across ROIs, indicative of poor overall model fit. Fig. 8 shows the normalized residuals for this subject, compared to another subject with lower residuals. In the retained sample, normalized residuals were low across all ROIs and did not systematically relate to MWF estimates (NAWM: *r* = −0.06, *p* = 0.795; CC: *r* = 0.01, *p* = 0.964; lesions: *r* = 0.35, *p* = 0.228), indicating that model fit quality did not bias MWF values in the analyzed dataset.

**Fig. 8:**
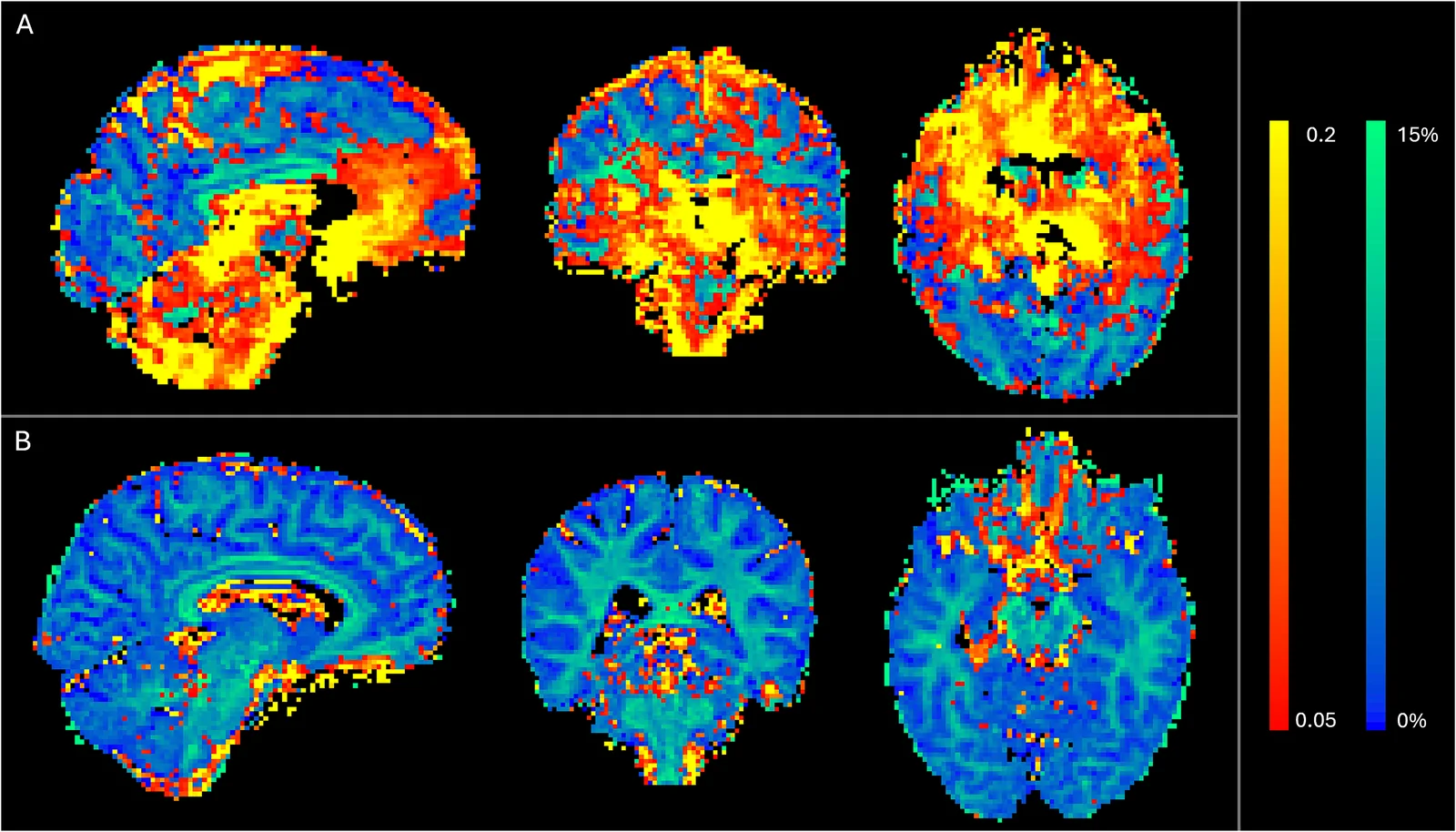
Representative myelin water fraction (MWF) maps (blue-green) and corresponding normalized residual maps (red-yellow) from two subjects, illustrating poor versus good fit quality. **(A)** Poor-quality example (male, SPMS, age 52.4 years, disease duration 18.9 years): normalized residuals exceeding 0.05 are distributed diffusely throughout the brain, indicating widespread model fitting error. **(B)** Good-quality example (male, RRMS, age 45.4 years, disease duration 21.3 years): normalized residuals exceeding 0.05 are largely confined to cerebrospinal fluid (CSF) and regions of reduced B0 field homogeneity, such as the orbitofrontal cortex.

## 4 Discussion

This study evaluated the optimization of myelin water imaging using a multi-echo gradient-echo sequence in people with MS and healthy controls, with the aim of identifying a protocol that balances scan efficiency with sensitivity to myelin water fraction.

The strong agreement between FA4 and FA5, reflected in the small bias and narrow limits of agreement observed in the Bland-Altman analysis across all ROIs, suggests that the fifth flip angle contributes little additional information while extending scan time. Reducing to FA3, however, led to systematic overestimation of MWF. Chan et al. (Chan et al., 2023) did not observe a bias in MWF values when comparing the subset of three flip angles [10,20,70] with the five flip angle dataset [5,10,20,50,70]. Of note, that comparison was based on MWI analysis without information from diffusion. In addition, we used a different distribution of flip angles in our FA5 protocol [10,20,30,40,70]. The reduction to three flip angles in our data probably reduced the accuracy of T1 estimation, a parameter critical for disentangling the myelin water signal from the intra- and extra-cellular water compartments in the three-compartment model (Chan et al., 2023).

The choice between resolutions is less straightforward. Reliability, as measured by the ICC-C was excellent for the FA4 and FA5 protocols, and remained good for the FA3 protocol, though it was somewhat lower for lesions at FA3. This indicates that MWF estimates at 2.0 mm and 2.5 mm resolutions are consistently ranked across subjects, even under a reduced flip angle scheme. However, consistency does not imply absolute agreement, which is demonstrated by the lower values in the ICC-AA. The Bland-Altman plots further show that MWF values at the two resolutions should not be treated as interchangeable in absolute terms. The two resolutions differed not only in spatial resolution, but also in TR, TE and total acquisition time. These parameter differences can each influence the estimated relaxation parameters in the model and the signal-to-noise ratio (SNR), which in turn affects the MWF estimations. After removal of poor quality voxels, volumes of the NAWM did not differ between resolutions, neither did model fit quality differ significantly between resolutions across any ROI. The observed differences in MWF values between resolutions therefore likely reflect inherent differences in SNR and voxel size, and small differences in acquisition parameters, rather than differences in model performance. Despite the reduced SNR inherent to smaller voxels, the 2.0 mm resolution maintained adequate signal quality for reliable MWF estimation, as shown by comparable model fit quality across resolutions. Although we assume that MWI will reflect (partly) other microstructural properties than DWI, the stronger associations between MWF and diffusion metrics observed at 2.0 mm suggest that this resolution may better capture microstructural variation in NAWM, potentially due to reduced partial volume effects. This is particularly relevant for lesion analyses, where smaller voxel sizes allow for more precise delineation of lesions. For these reasons, a 2.0 mm resolution may be preferable when the research question centers on lesion characterization or when sensitivity to microstructural associations is prioritized. That said, the 2.5 mm protocol demonstrated good MWF agreement and model fit across ROIs and remains a viable alternative in contexts where acquisition time is a limiting factor. When using the FA4 protocol, scan time of 2.5 mm is 3 min 20 s, thus 2 min 32 s shorter than the scan time of 2.0 mm (5 min 52 s).

Beyond technical optimization, the clinical findings provide insight into the sensitivity of MWF to disease-related myelin loss in MS. Contrary to expectations (Faizy et al., 2016), no differences in MWF in NAWM or corpus callosum were observed when comparing pwMS with healthy controls in this small cohort. When comparing subgroups, PPMS showed significantly lower NAWM MWF compared to RRMS and healthy controls, with a similar trend in the corpus callosum. This may reflect the more diffuse and progressive neurodegenerative processes associated with the primary progressive disease course, in which demyelination is thought to be less confined to discrete lesions and more widespread across the white matter (Miller and Leary, 2007). However, PPMS participants also had the highest median lesion volume and were on average older than the other subtypes, both of which may independently contribute to lower MWF. Higher lesion volume was associated with lower MWF not only within lesions, but also in NAWM and the corpus callosum. Overall, this pattern suggests that myelin loss in MS extends beyond lesions into tissue that appears normal on conventional MRI. Given the small group sizes, these findings should be considered exploratory and replicated in larger cohorts before drawing firm conclusions about subtype-specific differences in myelin content.

The model fit evaluation further highlighted the importance of quality control in myelin water imaging pipelines. One participant was excluded from all analyses due to consistently elevated normalized residuals across ROIs, suggesting a systematic failure of the model to adequately describe the signal. Possible factors that violate model assumptions include subject motion, hardware instability, or respiration-induced B0 fluctuations (Chan et al., 2023). These observations show that quality control on a per-subject basis is essential in the MWI workflow. Either a full dataset, or voxels with poor fit quality may warrant closer inspection before inclusion in further analyses. Moreover, the sensitivity of this MWI method also underscores the need to apply high-order shimming before acquisition.

Several limitations of this study should be acknowledged. The sample size was modest, particularly for subgroup analyses involving MS subtypes and lesion MWF, where smaller numbers reduced statistical power and resulted in wider confidence intervals. In particular, the RRMS, PPMS, and SPMS subgroups were small (*n* = 7, 4, and 3, respectively), and differences in age and lesion burden across subtypes cannot be fully disentangled from disease subtype in this cohort. These subtype comparisons should therefore be considered exploratory and should be validated in larger, age-balanced cohorts. Additionally, three participants (1 HC, 2 SPMS) were scanned using a 20-channel rather than a 64-channel head coil to accommodate larger head size; one of the SPMS patients was subsequently excluded from analysis for unrelated reasons (see Section 3.5), leaving 1 HC and 1 person with SPMS scanned with the 20-channel coil among the 24 participants in the final analysis. Given the small number of participants affected, a formal statistical comparison of coil type was not feasible; however, MWF and fit-quality values for these two participants fell within the range observed for the 64-channel group. Furthermore, multi-shell DWI data were obtained only at the lower resolution of 2.5 mm, and subsequently registered to MEGRE space at the spatial resolution of each protocol being processed. As a result, diffusion-derived parameters were upsampled when applied to the 2.0 mm MEGRE analyses. This asymmetry cannot be fully disentangled from genuine effects of MEGRE spatial resolution and should be considered when interpreting both the DIMWI-derived MWF estimates and the reported MWF–diffusion associations. Finally, the study was cross-sectional, precluding any conclusions about the reproducibility of MWF across repeated scans or its sensitivity to disease progression over time.

In conclusion, this study demonstrates that a FA4 MEGRE protocol offers an effective balance between scan efficiency and sensitivity to myelin content in MS. The choice between 2.0 mm and 2.5 mm resolution can be guided by the specific aims of the study. MWF successfully differentiated lesion tissue from NAWM, and lesion volume was associated with reduced myelin content not only within lesions but across broader white matter regions, supporting the presence of diffuse myelin damage in MS. Preliminary subtype-specific differences, with PPMS showing the lowest NAWM MWF, further highlighted the potential of MWF as a marker of disease burden. Monitoring model fit quality through normalized residuals is recommended as a routine quality control step. Future longitudinal studies with larger cohorts of pwMS are needed to establish the sensitivity of this optimized protocol to myelin changes over time.

## Acknowledgements and grand support

The authors acknowledge the Dutch MS Research Foundation (award number: 24–1225) for their support. This research has been executed within the MS Center Amsterdam, Amsterdam UMC.

## CRediT authorship contribution statement

**Ellis Donders:** Writing – review & editing, Writing – original draft, Visualization, Validation, Software, Project administration, Methodology, Investigation, Formal analysis, Data curation. **Guus H.J. Vorst:** Writing – review & editing, Methodology. **Pien M. Schipper:** Writing – review & editing, Data curation. **Joost P.A. Kuijer:** Writing – review & editing, Software, Methodology. **Eva M.M. Strijbis:** Writing – review & editing, Data curation. **Joep Killestein:** Writing – review & editing, Data curation. **Hugo Vrenken:** Writing – review & editing, Supervision. **Frederik Barkhof:** Writing – review & editing, Supervision, Conceptualization. **Menno M. Schoonheim:** Funding acquisition, Data curation, Conceptualization. **Petra J.W. Pouwels:** Writing – review & editing, Supervision, Software, Resources, Project administration, Methodology, Investigation, Funding acquisition, Data curation, Conceptualization.

## Declaration of competing interest

The authors declare the following financial interests/personal relationships which may be considered as potential competing interests:

E.M.M. Strijbis received speaker fees from Merck and Novartis.

J. Killestein received research grants for multicentre investigator initiated trials DOT-MS trial, ClinicalTrials. gov Identifier: NCT04260711 (ZonMW) and BLOOMS trial (ZonMW and Treatmeds), ClinicalTrials. gov Identifier: NCT05296161); received consulting fees for F. Hoffmann-La Roche, Biogen, Teva, Merck, Novartis and Sanofi/Genzyme (all payments to institution); reports speaker relationships with F. Hoffmann-La Roche, Biogen, Immunic, Teva, Merck, Novartis and Sanofi/Genzyme (all payments to institution); adjudication committee of MS clinical trial of Immunic (payments to institution only.

H. Vrenken: received research support from 10.13039/100004334Merck, Novartis, Pfizer, and Teva; consulting fees from Merck; and speaker honoraria from Novartis (all funds were paid to his institution).

F. Barkhof is part of the steering committee or Data Safety Monitoring Board member for Biogen, Merck, Eisai and Prothena. Advisory board member for Combinostics, Scottish Brain Sciences. Consultant for Roche, Celltrion, Rewind Therapeutics, Merck, Bracco. Research agreements with ADDI, Merck, Biogen, GE Healthcare, Roche. Co-founder and shareholder of Queen Square Analytics LTD.

M.M. Schoonheim serves on the editorial board of Neurology and Frontiers in Neurology; receives research support from the Dutch MS 10.13039/100005930Research Foundation, Eurostars-EUREKA, ARSEP, Amsterdam Neuroscience, MAGNIMS, and ZonMW (Vidi grant, project number 09150172010056); and has served as a consultant for or received research support from Atara Biotherapeutics, Biogen, Celgene/Bristol Meyers Squibb, EIP, Sanofi, MedDay, and Merck.

If there are other authors, they declare that they have no known competing financial interests or personal relationships that could have appeared to influence the work reported in this paper.

## Data Availability

Data will be made available on request.
