# Supplementary material for: Optimizing myelin water imaging in multiple sclerosis
Source: Neuroimage Clin. 2026 Nov 2;52:104080. doi: 10.1016/j.nicl.2026.104080 (PMC13769479; doi:10.1016/j.nicl.2026.104080)
Supplement: Supplementary file 1 [file mmc1.docx]

A. Supplementary

 **Figure A.1**. MWF Maps in sagittal, coronal and axial view of a pwMS. A. 2mm FA5; B. 2.5mm FA5; C. 2mm FA4; D. 2.5mm FA4; E. 2mm FA3; F. 2.5mm FA3.

**Figure A.2.** Scatter plots showing correlations of MWF values within corpus callosum across different FA measurements. Panels depict: FA4 vs. FA5 at 2 mm (A), FA3 vs. FA5 at 2 mm (B), FA3 vs. FA4 at 2.0 mm (C), FA4 vs. FA5 at 2.5 mm (D), FA3 vs. FA5 at 2.5 mm (E), and FA3 vs. FA4 at 2.5 mm (F). Abbreviations: MWF, myelin water fraction; CC, corpus callosum; FA, flip angle.

**Figure A.3.** Scatter plots showing correlations of MWF values within lesions across different FA measurements. Panels depict: FA4 vs. FA5 at 2 mm (A), FA3 vs. FA5 at 2 mm (B), FA3 vs. FA4 at 2.0 mm (C), FA4 vs. FA5 at 2.5 mm (D), FA3 vs. FA5 at 2.5 mm (E), and FA3 vs. FA4 at 2.5 mm (F). Abbreviations: MWF, myelin water fraction; FA, flip angle.

**Figure A.4.** Bland-Altman plots comparing MWF estimates in corpus callosum across flip angle protocols at two spatial resolutions. Abbreviation: CC: corpus callosum.

**Figure A.5.** Bland-Altman plots comparing MWF estimates in lesions in pwMS across flip angle protocols at two spatial resolutions.

**Figure A.6**. Bland-Altman plots comparing MWF estimates in corpus callosum across two spatial resolutions across different flip angle protocols. ΔMWF = MWF(2.0 mm) - MWF(2.5 mm).

**Figure A.7**. Bland-Altman plots comparing MWF estimates in lesions in pwMS across two spatial resolutions across different flip angle protocols. ΔMWF = MWF(2.0 mm) - MWF(2.5 mm).

**Figure A.8.** A. Subject's MWF map. B Overlay in red showing voxels where normalized residuals exceeded median+3IQR of this analysis, used to flag outliers/artifactual fits. C. Subject's MWF map after outlier voxels were excluded from the analysis.

**Table A1.** Normalized residuals in different regions of interests, in FA3, FA4, and FA5.

| ROI | Resolution | FA3 | FA4 | FA5 |
| --- | --- | --- | --- | --- |
| NAWM | 2.0 mm | 0.015 ± 0.006 | 0.016 ± 0.007 | 0.018 ± 0.008 |
|  | 2.5 mm | 0.016 ± 0.006 | 0.016 ± 0.007 | 0.017 ± 0.007 |
| Corpus Callosum | 2.0 mm | 0.022 ± 0.008 | 0.022 ± 0.009 | 0.024 ± 0.010 |
|  | 2.5 mm | 0.019 ± 0.009 | 0.020 ± 0.009 | 0.020 ± 0.009 |
| Lesions | 2.0 mm | 0.023 ± 0.009 | 0.025 ± 0.010 | 0.028 ± 0.011 |
|  | 2.5 mm | 0.023 ± 0.010 | 0.024 ± 0.009 | 0.025 ± 0.008 |
